# Cost-effectiveness of Maintenance Therapy Based on Molecular Classification Following Treatment of Primary Epithelial Ovarian Cancer in the United States

**DOI:** 10.1001/jamanetworkopen.2020.28620

**Published:** 2020-12-09

**Authors:** Courtney A. Penn, Melissa S. Wong, Christine S. Walsh

**Affiliations:** 1 Division of Gynecologic Oncology, Department of Obstetrics and Gynecology, Cedars-Sinai Medical Center, Los Angeles, California; 2 Division of Maternal Fetal Medicine, Department of Obstetrics and Gynecology, Cedars-Sinai Medical Center, Los Angeles, California

## Abstract

**Importance:**

There are large randomized clinical trials—SOLO-1 (Olaparib Maintenance Monotherapy in Patients With *BRCA* Mutated Ovarian Cancer Following First Line Platinum Based Chemotherapy [December 2018]), PRIMA (A Study of Niraparib Maintenance Treatment in Patients With Advanced Ovarian Cancer Following Response on Front-Line Platinum-Based Chemotherapy [September 2019]), and PAOLA-1 (Platine, Avastin and Olaparib in 1st Line [December 2019])—reporting positive efficacy results for maintenance regimens for women with primary, advanced epithelial ovarian cancer. The findings resulted in approval by the US Food and Drug Administration of the treatments studied as of May 2020. However, there are pressing economic considerations given the many eligible patients and substantial associated costs.

**Objective:**

To evaluate the cost-effectiveness of maintenance strategies for patients with (1) a *BRCA* variant, (2) homologous recombination deficiency without a *BRCA* variant, or (3) homologous recombination proficiency.

**Design, Setting, and Participants:**

In this economic evaluation of the US health care sector using simulated patients with primary epithelial ovarian cancer, 3 decision trees were developed, one for each molecular signature. The maintenance strategies evaluated were olaparib (SOLO-1), olaparib-bevacizumab (PAOLA-1), bevacizumab (PAOLA-1), and niraparib (PRIMA). Base case 1 assessed olaparib, olaparib-bevacizumab, bevacizumab, and niraparib vs observation of a patient with a *BRCA* variant. Base case 2 assessed olaparib-bevacizumab, bevacizumab, and niraparib vs observation in a patient with homologous recombination deficiency without a *BRCA* variant. Base case 3 assessed olaparib-bevacizumab, bevacizumab, and niraparib vs observation in a patient with homologous recombination proficiency. The time horizon was 24 months. Costs were estimated from Medicare claims, wholesale acquisition prices, and published sources. Probabilistic sensitivity analyses with microsimulation were then conducted to account for uncertainty and assess model stability. One-way sensitivity analyses were also performed. The study was performed from January through June 2020.

**Main Outcomes and Measures:**

Incremental cost-effectiveness ratios (ICERs) in US dollars per progression-free life-year saved (PF-LYS).

**Results:**

Assuming a willingness-to-pay threshold of $100 000/PF-LYS, none of the drugs could be considered cost-effective compared with observation. In the case of a patient with a *BRCA* variant, olaparib was the most cost-effective (ICER, $186 777/PF-LYS). The third-party payer price per month of olaparib would need to be reduced from approximately $17 000 to $9000 to be considered cost-effective. Olaparib-bevacizumab was the most cost-effective in the case of a patient with homologous recombination deficiency without a *BRCA* variant (ICER, $629 347/PF-LYS), and bevacizumab was the most cost-effective in the case of patient with homologous recombination proficiency (ICER, $557 865/PF-LYS). Even at a price of $0 per month, niraparib could not be considered cost-effective as a maintenance strategy for patients with homologous recombination proficiency.

**Conclusions and Relevance:**

The findings of this study suggest that, at current costs, maintenance therapy for primary ovarian cancer is not cost-effective, regardless of molecular signature. For certain therapies, lowering the drug price alone may not make them cost-effective.

## Key Points

**Question:**

Is maintenance therapy after first-line treatment of ovarian cancer cost-effective in the United States?

**Findings:**

In this economic evaluation of maintenance strategies after first-line ovarian cancer therapy, no maintenance regimen—including olaparib, olaparib-bevacizumab, bevacizumab, and niraparib—was cost-effective when using a willingness-to-pay threshold of $100 000 per progression-free life-year saved, even when stratified by molecular signatures. Olaparib monotherapy became cost-effective for patients with a *BRCA* variant when current olaparib pricing was reduced by half.

**Meaning:**

Various frontline maintenance therapies for ovarian cancer may have clinical benefit but may not be considered cost-effective, even with reductions in drug pricing.

## Teaser

This economic evaluation evaluated the cost-effectiveness of maintenance strategies for patients with (1) a *BRCA* variant, (2) homologous recombination deficiency without a *BRCA* variant, or (3) homologous recombination proficiency in the US health care sector using simulated patients with primary epithelial ovarian cancer.

## Introduction

Olaparib, a poly–(adenosine diphosphate–ribose) polymerase (PARP) inhibitor, achieved regulatory approval for maintenance therapy for women with newly diagnosed advanced ovarian cancer who carry a *BRCA* variant after publication of the SOLO-1 (Olaparib Maintenance Monotherapy in Patients With *BRCA* Mutated Ovarian Cancer Following First Line Platinum Based Chemotherapy) trial in December 2018, which showed a substantial improvement in progression-free survival with olaparib compared with placebo.^1^ The success of olaparib in staving off recurrent disease is an important advance because approximately 70% of women with advanced ovarian cancer relapse within 3 years after diagnosis, despite the absence of residual disease after primary surgery and platinum-based chemotherapy.^2^ Recurrent ovarian cancer, in turn, is typically incurable and associated with a median survival of 2 years.^3^

In the brief period since the publication of the SOLO-1 trial, there have been additional large randomized clinical trials—PRIMA (A Study of Niraparib Maintenance Treatment in Patients With Advanced Ovarian Cancer Following Response on Front-Line Platinum-Based Chemotherapy [September 2019]), VELIA (Veliparib With Carboplatin and Paclitaxel and as Continuation Maintenance Therapy in Adults With Newly Diagnosed Stage III or IV, High-grade Serous, Epithelial Ovarian, Fallopian Tube, or Primary Peritoneal Cancer [December 2019]), and PAOLA-1 (Platine, Avastin and Olaparib in 1st Line [December 2019])—that have reported positive efficacy results for various maintenance regimens involving PARP inhibitors following first-line treatment of newly diagnosed ovarian cancer, stratified by *BRCA* and homologous recombination status.^4,5,6^ With the exception of the VELIA trial, which examined the efficacy of veliparib, the drugs studied (olaparib, niraparib, and bevacizumab) in the aforementioned randomized clinical trials are commercially available and, as of May 2020, have achieved regulatory approval through the US Food and Drug Administration (FDA). This leaves clinicians with several options for maintenance regimens for the approximately 22 000 women who have receive a diagnosis of advanced ovarian cancer in the US annually.^7^

Notwithstanding the clinical promise of the various strategies considered in these trials, economic evaluation is critical given the large number of newly eligible patients for the studied therapies in addition to the cost associated with treatments. Cost-effectiveness analyses could help assess whether the treatment benefit afforded is of justifiable cost. To our knowledge, there has not been a peer-reviewed cost-effectiveness analysis study published that compares the first-line maintenance regimens in these trials. The aim of our study was to evaluate the cost-effectiveness of the maintenance strategies outlined in the SOLO-1 (olaparib), PRIMA (niraparib), and PAOLA-1 (olaparib-bevacizumab, and bevacizumab) trials for patients with a *BRCA* variant, patients with homologous recombination deficiency without a *BRCA* variant, and patients with homologous recombination proficiency (note that the veliparib regimen from the VELIA trial was not assessed because this drug was not filed for approval at the time of our analysis).

## Methods

This study was conducted from January through June 2020 in accordance with the Consolidated Health Economic Evaluation Reporting Standards (CHEERS) reporting guideline.^8^ It is deemed exempt from institutional review board review because publicly available information was used.

### Decision Models

**Figure 1.**
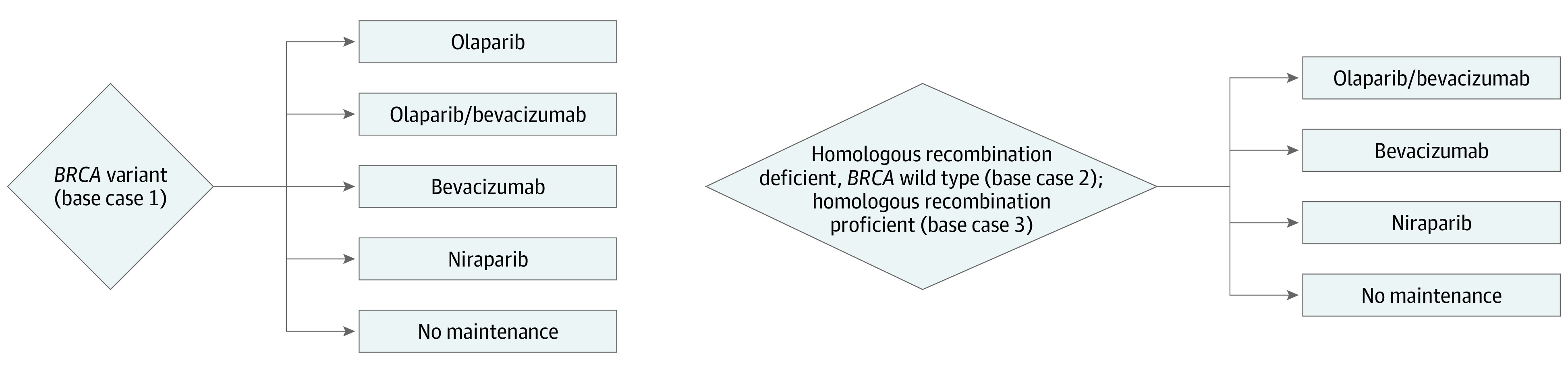
Summary Schematic of Treatment Strategies for Base Case 1 (A), Base Case 2 (B), and Base Case 3 (C)

A hypothetical patient with newly diagnosed, advanced (stage III or IV) epithelial ovarian cancer who had already undergone cytoreductive surgery and had a complete or partial response to platinum-based chemotherapy was eligible for entrance into 1 of 3 models: one for patients with a *BRCA* variant, one for patients with homologous recombination deficiency without a *BRCA* variant, or one for patients with homologous recombination proficiency. Base case 1 followed a patient with a *BRCA* variant through 5 possible frontline maintenance strategies: olaparib, olaparib-bevacizumab, bevacizumab, niraparib, and no maintenance therapy. Base cases 2 and 3 followed a patient with homologous recombination deficiency without a *BRCA* variant and a patient with homologous recombination proficiency, respectively, through 4 possible frontline maintenance regimens: olaparib-bevacizumab, bevacizumab, niraparib, and no maintenance therapy (Figure 1). These models were based on the study designs from the SOLO-1, PRIMA, and PAOLA-1 trials.^1,4,5^ Given that the outcomes reported in these trials were limited to progression-free survival times and Kaplan-Meier progression-free survival curves, Markov modeling was not used to avoid potentially unrealistic modeling assumptions. The fraction of patients in a progression-free state vs a disease state was estimated over the model’s time horizon using the Kaplan-Meier curves. This method of cost-effectiveness modeling has been described and validated elsewhere.^9^

The treatment regimens included in the model were derived from the SOLO-1, PRIMA, and PAOLA-1 treatment groups^1,4,5^ (eTable 1 in the Supplement). Given that 71% of patients in the PRIMA trial required dose reduction from 300 mg to 200 mg daily,^4^ it was assumed that 71% of patients in the model were taking 200 mg daily and that 29% of patients were taking 300 mg daily. In calculating bevacizumab dosing, body weight was assumed to be 70 kg. Although these trials examined different randomized populations, the key patient demographic and clinical characteristics were similar overall, allowing for a post hoc comparison via cost-effectiveness analysis (eTable 2 in the Supplement). One notable difference is that the PRIMA trial excluded patients with stage III optimally cytoreduced cancer after primary debulking, whereas the other trials allowed enrollment of patients with stage III cancer who underwent primary optimal cytoreduction. However, among all patients who had cytoreductive surgery in the PRIMA trial, 50% achieved no macroscopic residual disease.^10^ Moreover, all of the patients enrolled in these trials had to have a complete or partial response to platinum-based chemotherapy, making the disease burden at the time of initiation of maintenance treatment similar between trials.

Patients receiving bevacizumab (base cases 2 and 3) were assumed to have had 5 cycles of bevacizumab prior to entrance into the model. The time horizon was limited to 24 months given the absence of overall survival data from the trials. In addition, this time horizon approximated the mean follow-up period of the trials from which the treatment groups were derived. No discounting or drug discontinuation was accounted for given the limited time horizon.

### Statistical Analysis

#### Health State Utilities

Incremental cost-effectiveness ratios (ICERs) were calculated in US dollars per progression-free life-year saved (PF-LYS), as has been done in other economic evaluations of oncology drugs lacking overall survival data.^11,12^ Imputing overall survival estimates in the absence of overall survival data results in a model with significant uncertainty because patients with ovarian cancer tend to have highly variable treatment courses after recurrence. Because quality-of-life end points were assessed differently among the trials, quality-adjusted life-years were not estimated. Kaplan-Meier estimates of progression-free survival from the randomized clinical trials (eTable 3 in the Supplement) were used to determine the probability of being disease free at 24 months. The gain in progression-free survival in months was then multiplied by the 24-month progression-free survival projection to calculate PF-LYS. A willingness-to-pay (WTP) threshold of $100 000/PF-LYS was used to guide the interpretation of ICERs.

#### Costs

Costs were considered from a US health care perspective. Costs included those associated with drug acquisition, administration, monitoring, and adverse effects. The 2020 RED BOOK wholesale acquisition costs were used to estimate the prices of monthly supplies of olaparib and niraparib.^13^ The unit bevacizumab cost was estimated using the Centers for Medicare & Medicaid Services April 2020 Average Sales Price Drug Pricing Files.^14^ Administration (in the case of bevacizumab) and monitoring costs were obtained from the 2020 Medicare Physician Fee Schedule.^15^ Patients receiving a PARP inhibitor were assumed to require a complete blood count weekly for the first month, followed by a complete blood count monthly. It was assumed that, for these patients, office visits occurred monthly and a cancer antigen 125 test every 3 months. The cost of 15 comprehensive metabolic panels was added to the aforementioned PARP inhibitor costs for patients receiving combination olaparib-bevacizumab. For patients receiving bevacizumab alone, it was assumed that they had a complete blood count, a comprehensive metabolic panel, and an office visit (including a urine dipstick) with each infusion and a cancer antigen 125 test every 3 months. It was assumed that patients without a maintenance regimen had an office visit and cancer antigen 125 test every 3 months. Routine imaging was not incorporated into monitoring costs. Costs for treating adverse events that occurred in 20% or more of the respective study population and had previously been quantified^16^ were considered in the model. It was assumed that adverse events occurred independently and that the probability of those events remained constant over the 24-month time horizon. This assumption was made because adverse events were reported in the trials as a sum of each complication among participants throughout the total trial period. Germline and somatic genetic testing costs were not included because it was assumed that genetic testing was performed at the time of diagnosis, prior to entrance into our model. Detailed cost inputs used in the model are outlined in eTable 4 in the Supplement.

#### Sensitivity Analyses

Using TreeAge Pro 2019 R1, we performed 1-way deterministic sensitivity analyses on key model parameters in all 3 base cases to account for uncertainty. The key parameters varied and included drug and adverse event costs, progression-free survival advantage, and 24-month progression-free survival probability. These parameters were tested individually to determine the required threshold, if possible, at which the associated treatment regimen would become cost-effective using a WTP threshold of $100 000/PF-LYS. In addition, each variable was tested within realistic ranges (cost parameters altered by ±50% and progression-free survival probabilities altered by ±10%), and the resulting ICERs were calculated.

Probabilistic sensitivity analyses with microsimulation were performed for each base case within the following parameter distributions: olaparib, bevacizumab, and niraparib monthly price ±50%; olaparib, bevacizumab, and niraparib costs associated with adverse events ±50%; progression-free survival advantage compared with no maintenance for each possible treatment regimen ±10%; and 24-month progression-free survival probability for each possible treatment regimen ±10% (1000 trials were simulated with 1000 patients).

## Results

### Incremental Cost-effectiveness Ratios

The 24-month estimated costs of the studied treatment regimens were as follows: $418 848 for olaparib, $545 758 for olaparib-bevacizumab, $133 591 for bevacizumab, and $492 226 for niraparib. The absence of maintenance therapy was estimated to have a 24-month cost of $3051. In base case 1 (*BRCA* variant), the incremental PF-LYS compared with no maintenance was 2.23 for olaparib, 1.48 for olaparib-bevacizumab, 0.26 for bevacizumab, and 0.46 for niraparib. In base case 2 (homologous recombination deficient with a *BRCA* variant), the incremental PF-LYS compared with no maintenance was 0.86 for olaparib-bevacizumab, 0.18 for bevacizumab, and 0.46 for niraparib. In base case 3 (homologous recombination proficient), the incremental PF-LYS compared with no maintenance was 0.25 for olaparib-bevacizumab, 0.23 for bevacizumab, and 0.05 for niraparib. The resulting ICERs for each base case revealed that none of the treatment options were cost-effective at the prespecified WTP threshold. However, olaparib was the most cost-effective maintenance option in base case 1 (ICER, $186 777/PF-LYS) (Table 1), olaparib-bevacizumab was the most cost-effective maintenance option in base case 2 (ICER, $629 347/PF-LYS) (Table 1), and bevacizumab was the most cost-effective maintenance option in base case 3 (ICER, $557 865/PF-LYS) (Table 1).

**Table 1.** Summary Base Case Results

Outcome	No maintenance	Olaparib	Olaparib-bevacizumab	Bevacizumab	Niraparib
*BRCA* variant					
24-Month cost, $	3051	418 848	545 758	133 591	492 226
24-Month incremental cost, $	[Referent]	415 798	542 708	130 541	489 176
24-Month incremental PF-LYS	[Referent]	2.23	1.48	0.26	0.46
ICER/PF-LYS, $	[Referent]	186 777	366 199	508 434	1 069 627
Homologous recombination deficient, *BRCA* wild type					
24-Month cost, $	3051	Not studied	545 758	133 591	492 226
24-Month incremental cost, $	[Referent]	Not studied	542 708	130 541	489 176
24-Month incremental PF-LYS	[Referent]	Not studied	0.86	0.18	0.46
ICER/PF-LYS, $	[Referent]	Not studied	629 347	717 255	1 072 754
Homologous recombination proficient					
24-Month cost, $	3051	Not studied	545 758	133 591	492 226
24-Month incremental cost, $	[Referent]	Not studied	542 708	130 541	489 176
24-Monoth incremental PF-LYS	[Referent]	Not studied	0.25	0.23	0.05
ICER/PF-LYS, $	[Referent]	Not studied	2 153 600	557 865	10 870 576

Abbreviations: ICER, incremental cost-effectiveness ratio; PF-LYS, progression-free life-years saved.

### Sensitivity Analyses

On probabilistic sensitivity analyses with 1000 simulated patients, olaparib remained the most cost-effective option in base case 1 (*BRCA* variant), olaparib-bevacizumab therapy remained the most cost-effective option in base case 2 homologous recombination deficient without a *BRCA* variant), and bevacizumab remained the most cost-effective option in base case 3 (homologous recombination proficient). When the inputs for base case 1 were concurrently varied, olaparib monotherapy yielded an ICER less than or equal to the WTP threshold in 10/1000 patients (1%) compared with no maintenance treatment. No treatment regimens in base case 2 or 3 yielded ICERs at or below the WTP threshold compared with no maintenance in the 1000 trials.

**Figure 2.**
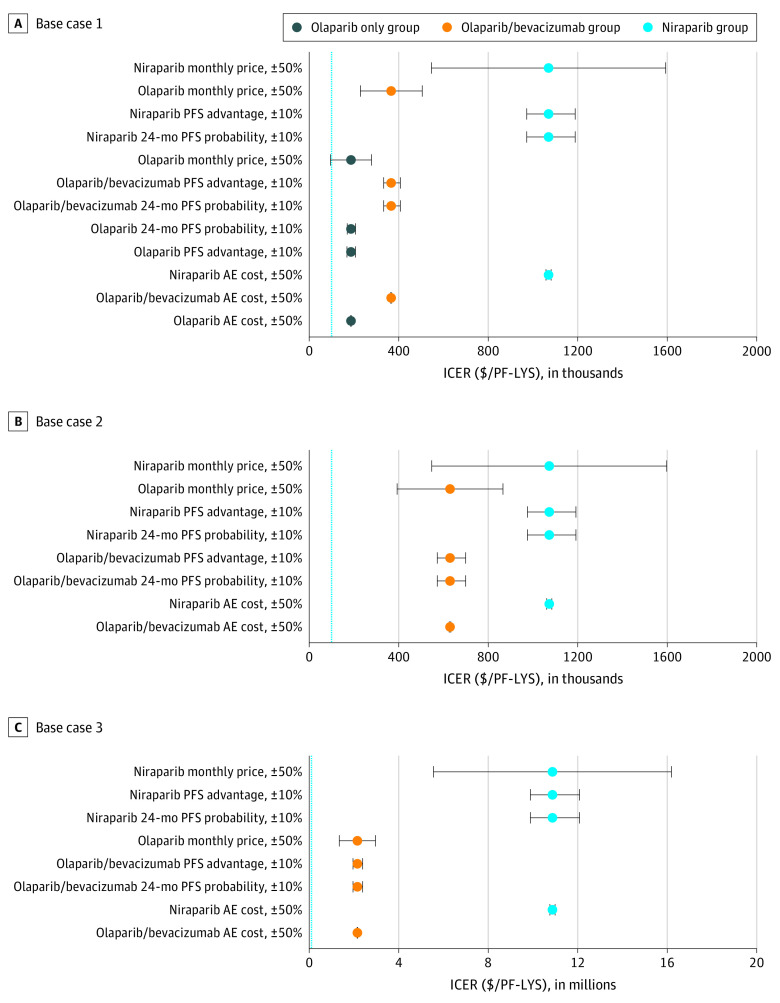
Deterministic Sensitivity Analyses for Base Case 1 (*BRCA* variant), Base Case 2 (Homologous Recombination Deficient, *BRCA* Wild Type), and Base Case 3 (Homologous Recombination Proficient) AE indicates adverse event; ICER, incremental cost-effectiveness ratio; PF-LYS, progression-free life-years saved; PFS, progression-free survival.

On 1-way sensitivity analysis, olaparib monotherapy in base case 1 (*BRCA* variant) became cost-effective at the WTP threshold when the average wholesale acquisition price of olaparib was reduced 47% from $16 999 to approximately $8950 per month (Table 2). When other key parameters within realistic ranges (costs ±50% and progression-free survival utilities and probabilities ±10%) were varied in 1-way sensitivity analyses (Figure 2A), no other treatment strategy aside from olaparib monotherapy became cost-effective at the WTP threshold.

**Table 2.** Cost-effective Threshold Values From Deterministic Sensitivity Analysis

Variable	Input value	Cost-effective value
Niraparib price per monthly cycle, $		
*BRCA* variant	19 947	1470
Homologous recombination deficiency, *BRCA* wild type	19 947	1465
Homologous recombination proficiency	19 947	NR
Olaparib price per monthly cycle, $		
Olaparib-only regimen (patients with a *BRCA* variant)	16 999	8950
Olaparib-bevacizumab regimen		
*BRCA* variant	16 999	435
Homologous recombination deficiency, *BRCA* wild type	16 999	NR
Homologous recombination proficiency	16 999	NR
Olaparib-bevacizumab combined monthly cost, $		
*BRCA* variant	22 249	5811
Homologous recombination deficiency, *BRCA* wild type	22 249	3230
Homologous recombination proficiency	22 249	686
**Adverse event cost, $**		
Niraparib		
*BRCA* variant	10 447	NR
Homologous recombination deficiency, *BRCA* wild type	10 447	NR
Homologous recombination proficiency	10 447	NR
Olaparib		
*BRCA* variant	7817	NR
Olaparib-bevacizumab		
*BRCA* variant	6430	NR
Homologous recombination deficiency, *BRCA* wild type	6430	NR
Homologous recombination proficiency	6430	NR
**PFS advantage, y**		
Niraparib		
*BRCA* variant	0.93	9.98
Homologous recombination deficiency, *BRCA* wild type	0.95	10.19
Homologous recombination proficiency	0.23	24.46
Olaparib		
*BRCA* variant	3.01	5.62
Olaparib-bevacizumab		
*BRCA* variant	1.95	7.14
Homologous recombination deficiency, *BRCA* wild type	1.66	10.44
Homologous recombination proficiency	0.93	20.10
**24-Month PFS probability**		
Niraparib		
*BRCA* variant	0.49	NR
Homologous recombination deficiency, *BRCA* wild type	0.48	NR
Homologous recombination proficiency	0.2	NR
Olaparib		
*BRCA* variant	0.74	NR
Olaparib-bevacizumab		
*BRCA* variant	0.76	NR
Homologous recombination deficiency, *BRCA* wild type	0.52	NR
Homologous recombination proficiency	0.27	NR

Abbreviations: NR, not reached at plausible value (eg, cost of $0, probability of 100%); PFS, progression-free survival.

Regarding base case 2 (homologous recombination deficient without a *BRCA* variant), no therapy could be considered cost-effective at the WTP threshold when individual variables were altered within plausible ranges (Figure 2B). However, olaparib-bevacizumab therapy could be considered cost-effective if the average monthly price of both drugs was reduced from $22 249 to $3230, an 85% decrease from current pricing (Table 2). Alternatively, olaparib-bevacizumab would need to have a 10.4-year progression-free survival advantage compared with no maintenance—a 6.3-fold increase from its current 1.7-year progression-free survival advantage—to be considered cost-effective at current pricing.

Similarly, in base case 3 (homologous recombination proficient), no therapy could be considered cost-effective at the WTP threshold when parameters were varied plausibly (Figure 2C). At a cost of $0 per month, niraparib—the maintenance drug currently approved by the FDA for the homologous recombination proficient population—could not be considered cost-effective compared with no maintenance in this population (Table 2). At current costs, niraparib could be considered cost-effective if the progression-free survival advantage compared with no maintenance was 24.5 years, a 108.7-fold increase from the current progression-free survival advantage of 0.23 years.

For all cases, alterations in the niraparib monthly drug price—from 50% ($9974) to 150% ($29 921) its current cost—varied the resulting ICERs most significantly. When the price of niraparib was altered within this range, the difference between the upper and lower limits of the ICER of niraparib compared with no treatment was $1 046 784/PF-LYS in base case 1, $1 049 845/PF-LYS in base case 2, and $10 638 427/PF-LYS in base case 3 (Figure 2). The ICERs of all treatment regimens for each base case were least sensitive to fluctuations in the costs associated with adverse events.

## Discussion

Compared with observation, this study demonstrates that currently available frontline maintenance therapies for women with primary, advanced epithelial ovarian cancer are not cost-effective, regardless of molecular signature, in our cost-effectiveness model. One-way sensitivity analyses showed that price reductions would make some of these therapies cost-effective, as in the case of olaparib for women with a *BRCA* variant, which would become cost-effective if the current monthly pricing were reduced by half. However, not all therapies would become cost-effective even if the medication were free, as in the case of niraparib for patients with homologous recombination proficiency. Such findings provide a perspective from which to interpret the positive efficacy results of the SOLO-1, PAOLA-1, and PRIMA trials.

As a result of the progression-free survival advantage found with the various maintenance regimens in the SOLO-1, PAOLA-1, and PRIMA trials, the FDA has issued approvals for maintenance therapy in specific molecular subgroups of patients with ovarian cancer: olaparib for patients with a *BRCA* variant (December 19, 2018)^17^; olaparib combined with bevacizumab for patients with homologous recombination deficiency (May 8, 2020)^18^; and niraparib for patients regardless of molecular signature, including patients with homologous recombination proficiency (April 29, 2020).^19^ The approvals may lead to reflexive use of these treatments for frontline maintenance for all patients without the current cost being commensurate with the effectiveness. The potential financial toxicity that could result, combined with the lack of long-term follow-up and overall survival data from these studies, may have a cumulative net negative patient and societal impact.

For example, GOG 218 (Carboplatin and Paclitaxel With or Without Bevacizumab in Treating Patients With Stage III or Stage IV Ovarian Epithelial, Primary Peritoneal, or Fallopian Tube Cancer) and ICON7 [A Randomised, Two-Arm, Multicentre Gynaecologic Cancer InterGroup Trial of Adding Bevacizumab to Standard Chemotherapy (Carboplatin and Paclitaxel) in Patients With Epithelial Ovarian Cancer] were 2 large phase 3 trials that demonstrated a progression-free survival advantage in short-term follow-up with frontline bevacizumab maintenance for patients with stage III or IV (GOG 218) and patients with stage I to IV (ICON7) ovarian cancer.^20,21^ However, this progression-free survival benefit was lost in long-term follow-up of the ICON7 cohort, and neither study ultimately showed an overall survival advantage except for specific populations with high-risk disease. Several economic evaluations prior to the publication of overall survival data also revealed that maintenance bevacizumab in the upfront setting had relatively low value.^22,23^ For example, a 2015 cost-utility analysis of the GOG 218 bevacizumab regimen for patients with ovarian cancer reported an ICER of $632 571/PF-LYS compared with no maintenance (relatively similar to the mean ICER for bevacizumab maintenance across all base cases found in our study, $594 518/PF-LYS, considering our study did not include bevacizumab costs during adjuvant chemotherapy).^22^ With added quality-of-life data, the ICER increased to $792 380/quality-adjusted progression-free year. Taken in isolation, the progression-free survival advantage seen in the short-term follow-up reported initially in the GOG 218 and ICON7 trials would suggest that the standard of care for patients with newly diagnosed ovarian cancer should be adjuvant and maintenance bevacizumab therapy; however, the added cost and overall survival information described makes the role of maintenance bevacizumab in the upfront setting less certain, as noted by the American Society of Clinical Oncology Gynecologic Oncology Education Committee.^24^ The efficacy of frontline maintenance PARP inhibitor treatment should be viewed in a similar context, taking into account the known benefits, harms, and costs.

This does not imply that maintenance regimens be withheld from patients with ovarian cancer in the upfront setting simply because they are not cost-effective—rather, the effectiveness of maintenance therapy should be interpreted within the context of its expense. For example, olaparib maintenance therapy for patients with a *BRCA* variant has an ICER greater than the accepted $100 000/PF-LYS WTP threshold (ICER, $186 777/PF-LYS), but it could realistically become cost-effective with a reduction in olaparib pricing by 47%. The 3-year progression-free survival advantage seen in patients with a *BRCA* variantwho took maintenance olaparib in the SOLO-1 trial thus likely warrants clinical use in that population. Conversely, the use of niraparib in the homologous recombination proficiency population may be less certain. The ICER was substantially above the WTP threshold ($10 870 576/PF-LYS), and niraparib did not become cost-effective even when its price was reduced to $0 per month. The 3-month progression-free survival advantage in the homologous recombination proficient population with maintenance niraparib thus may not warrant the cost, especially in the absence of long-term and overall survival data.

### Limitations

An inherent limitation of our analysis is that it relies on cross-trial comparison between 3 randomized clinical trials, which is necessary in the absence of a head-to-head randomized clinical trial comparing all maintenance regimens. Although the study populations of these trials had similar characteristics (eTable 2 in the Supplement) and the patients were required to have complete or partial response following chemotherapy, there undoubtedly is between-trial variability for which we cannot account. An attempt was made to mitigate interstudy heterogeneity by using the control groups of the trials as reference when possible. Another limitation is that the utility and probability estimates for the molecular signatures (*BRCA* variant, homologous recombination deficient, and homologous recombination proficient) were derived from subgroup analyses, which the trials were not powered to perform. The uncertainty in these values was accounted for in our probabilistic sensitivity analyses that varied parameters liberally. The stability of our findings from those analyses suggests that there is a low probability of misinterpreting the relative cost-effectiveness of the treatments studied.

## Conclusions

Currently available frontline maintenance therapies for advanced, epithelial ovarian cancer cannot be considered cost-effective using a WTP threshold of $100 000/PF-LYS. Maintenance olaparib for patients with a *BRCA* variant could feasibly become cost-effective if its price were reduced by half. Other maintenance strategies, such as niraparib for patients with homologous recombination proficiency, do not have obvious pragmatic solutions like price reduction to make them sufficiently cost-effective and may require further investigation to allow for a more certain and complete interpretation of the efficacy results before use in clinical settings.

## Notes

Supplement. **eTable 1.** Treatment Regimens **eTable 2.** Patient Demographic/Characteristic Comparison Among Cohorts From the SOLO-1, PRIMA, and PAOLA-1 Trials **eTable 3.** Progression-Free Survival Estimates **eTable 4.** Model Cost (USD) Parameters **eReferences.**
