# Supplementary material for: Cost-effectiveness of Maintenance Therapy Based on Molecular Classification Following Treatment of Primary Epithelial Ovarian Cancer in the United States
Source: JAMA Netw Open. 2020 Dec 9;3(12):e2028620. doi: 10.1001/jamanetworkopen.2020.28620 (PMC7726632; doi:10.1001/jamanetworkopen.2020.28620)
Supplement: Supplement. — eTable 1. Treatment Regimens eTable 2. Patient Demographic/Characteristic Comparison Among Cohorts From the SOLO-1, PRIMA, and PAOLA-1 Trials eTable 3. Progression-Free Survival Estimates eTable 4. Model Cost (USD) Parameters eReferences. [file jamanetwopen-e2028620-s001.pdf]

Supplemental Online Content

Penn CA, Wong MS, Walsh CS. Cost-effectiveness of maintenance therapy based on molecular classification following treatment of primary epithelial ovarian cancer in the United States. *JAMA Netw Open*. 2020;3(12):e2028620.
doi:10.1001/jamanetworkopen.2020.28620

eTable 1. Treatment Regimens
eTable 2. Patient Demographic/Characteristic Comparison Among Cohorts From the SOLO-1, PRIMA, and PAOLA-1 Trials
eTable 3. Progression-Free Survival Estimates
eTable 4. Model Cost (USD) Parameters
eReferences.

This supplemental material has been provided by the authors to give readers additional information about their work.

eTable 1. Treatment Regimens

Drug	Dose	Cycle Length	No of Cycles
Olaparib	300 mg BID	30 days	24
Niraparib	200-300 mg QD	30 days	24
Olaparib/ Bevacizumab	olaparib 300 mg BID; bevacizumab 15 mg/kg	olaparib 30 days; bevacizumab 21 days	olaparib 24; bevacizumab 15[a]
Bevacizumab	15 mg/kg	21 days	15[a]

[a] It was assumed that patients receiving bevacizumab had five bevacizumab cycles with platinum-based therapy prior to entering the model.

© 2020 Penn CA et al. *JAMA Network Open.*

eTable 2. Patient demographic/characteristic comparison among cohorts from the SOLO-1[1], PRIMA[2], and PAOLA-1[3] trials

	SOLO-1	PRIMA	PAOLA-1	
**Characteristic**	**Olaparib**	**Niraparib**	**Olaparib/ Bevacizumab**	**Bevacizumab**
Median age, years				
*BRCA*mut	53	NR	57	56
HRd	-	58	58	58
Overall	-	62	61	60
Eastern Cooperative Oncology Group (ECOG) performance status 0, %				
*BRCA*mut	77	NR	73	70
HRd	-	74	75	76
Overall	-	69	70	70
Serous histology, %				
*BRCA*mut	95	NR	96	91
HRd	-	95	95	94
Overall	-	96	97	94
FIGO stage III, %				
*BRCA*mut	85	NR	71	68
HRd	-	65	71	68
Overall	-	65	70	69
No residual disease after cytoreduction, %				
*BRCA*mut	78	NR	69	71
HRd	-	NR	68	65
Overall	-	50	65	65
Complete response after platinum-based chemotherapy, %				
*BRCA*mut	82	NR	81	76
HRd	-	75	79	74
Overall	-	69	74	72
Abbreviations:				
*BRCA*mut, *BRCA* mutant. Includes patients with *BRCA1* and *BRCA2* mutations.				
HRd, homologous-recombination deficiency. Includes HRD positive patients with a *BRCA* mutation and without a *BRCA* mutation.				
NR, not reported.				

© 2020 Penn CA et al. *JAMA Network Open.*

eTable 3. Progression-free survival estimates

Regimen	24 month disease-free/survival probability	Survival advantage (months)	Source(s)
*BRCA*mut			
Olaparib monotherapy	0.74	36.1	SOLO-1
Niraparib monotherapy	0.49	11.2	PRIMA
Olaparib + Bevacizumab	0.76	23.4	PAOLA-1, SOLO-1 (referent)
Bevacizumab monotherapy	0.39	7.9	PAOLA-1, SOLO-1 (referent)
No maintenance	0.35	0	SOLO-1 (referent)
HRd, *BRCA*wt			
Niraparib monotherapy	0.48	11.4	PRIMA
Olaparib + Bevacizumab	0.52	19.9	PAOLA-1, PRIMA (referent)
Bevacizumab monotherapy	0.26	8.4	PAOLA-1, PRIMA (referent)
No maintenance	0.3	0	PRIMA (referent)
HRp			
Niraparib monotherapy	0.2	2.7	PRIMA
Olaparib + Bevacizumab	0.27	11.2	PAOLA-1, PRIMA (referent)
Bevacizumab monotherapy	0.26	10.8	PAOLA-1, PRIMA (referent)
No maintenance	0.15	0	PRIMA (referent)
Abbreviations:			
*BRCA*mut, *BRCA* mutant.			
*BRCA*wt, *BRCA* wild type.			
HRd, homologous recombination deficiency.			
HRp, homologous recombination proficiency.			

© 2020 Penn CA et al. *JAMA Network Open.*

eTable 4. Model cost (USD) parameters

Variable	Input Value	Source
Bevacizumab		
Price per mg	8	CMS April 2020 ASP Drug Pricing File, accessed May 22, 2020[4]
Price per 3-week cycle[a]	8 400	
Price for 15 cycles	126 000	
Niraparib		
Price per 100 mg pill	290	AWP, accessed May 22, 2020[5]
Price per monthly cycle[b]	19 947	
Price for 24 months	478 729	
Olaparib		
Price per 150 mg pill	142	AWP, accessed May 22, 2020[5]
Price per monthly cycle	16 999	
Price for 24 months	407 981	
Oral drug administration		
Bevacizumab administration per hour	143	HCPCS 96413, CMS.gov , accessed May 22, 2020[6]
Price for 15 cycles of bevacizumab	2 138	
Monitoring costs		
Level 4 office visit	110	HCPCS 99214, CMS.gov, accessed May 22, 2020[6]
CA 125	21	HCPCS 86304, CMS.gov, accessed May 22, 2020[6]
CBC with differential	8	HCPCS 85025, CMS.gov, accessed May 22, 2020[6]
CMP	11	HCPCS 80053, CMS.gov, accessed May 22, 2020[6]
CT abdomen/pelvis	332	HCPCS 74177, CMS.gov, accessed May 22, 2020[6]
Bevacizumab monitoring cost, 15 cycles	2 035	
Per cycle cost	136	
Additional year of regular monitoring after 15 cycles	525	
Monthly bev only monitoring costs	107	
Total bev only monitoring costs	2 560	
PARP inhibitor monitoring cost, 24 months	3 050	
Per month cost	127	
PARP + bevacizumab monitoring, 24 months	3 208	
No maintenance monitoring, 24 months	1 050	
Per month cost	44	
Adverse events		Wong, et al., 2018[7]

© 2020 Penn CA et al. *JAMA Network Open.*

Variable	Input Value	Source
Bevacizumab	2 892	
Olaparib	7 817	
Niraparib	10 447	
Olaparib + bevacizumab	6 430	
No maintenance	2 000	
[a] Average patient weight assumed to be 70 kg. Bevacizumab dose assumed to be 15 mg/kg every three weeks.		
[b] Assumed 71% of patients taking 200 mg/day, given dose reduction from 300 mg/day in 71% of patients in PRIMA study.		
Abbreviations:		
CMS, Centers for Medicare & Medicaid Services		
AWP, Average Wholesale Price		
CBC, Complete Blood Count		
CMP, Comprehensive Metabolic Panel		

© 2020 Penn CA et al. *JAMA Network Open.*

eReferences:

1. Moore K, Colombo N, Scambia G, et al. Maintenance Olaparib in Patients with Newly Diagnosed Advanced Ovarian Cancer. *N Engl J Med.* 2018;379(26):2495-2505.
2. Gonzalez-Martin A, Pothuri B, Vergote I, et al. Niraparib in Patients with Newly Diagnosed Advanced Ovarian Cancer. *N Engl J Med.* 2019;381(25):2391-2402.
3. Ray-Coquard I, Pautier P, Pignata S, et al. Olaparib plus Bevacizumab as First-Line Maintenance in Ovarian Cancer. *N Engl J Med.* 2019;381(25):2416-2428.
4. Centers for Medicare and Medicaid Services. April 2020 ASP drug pricing files. https://www.cms.gov/apps/ama/license.asp?file=https%3A//www.cms.gov/files/zip/april-2020-asp-pricing-file.zip Accessed May 22, 2020.
5. Truven Health Analytics, IBM Watson. Micromedex RED BOOK Database. Available at: http://truvenhealth.com/Products/Micromedex/Product-Suites/Clinical-Knowledge/RED-BOOK. Accessed May 22, 2020.
6. Centers for Medicare & Medicaid Services. Medicare physician fee schedule look-up tool. Year 2020. Available at: https://www.cms.gov/apps/physician-fee-schedule/overview.aspx. Accessed May 22, 2020. .
7. Wong W, Yim YM, Kim A, et al. Assessment of costs associated with adverse events in patients with cancer. *PloS one.* 2018;13(4):e0196007-e0196007.

© 2020 Penn CA et al. *JAMA Network Open.*
